# An Update on Statistical Boosting in Biomedicine

**DOI:** 10.1155/2017/6083072

**Published:** 2017-08-02

**Authors:** Andreas Mayr, Benjamin Hofner, Elisabeth Waldmann, Tobias Hepp, Sebastian Meyer, Olaf Gefeller

**Affiliations:** ^1^Institut für Medizininformatik, Biometrie und Epidemiologie, Friedrich-Alexander-Universität Erlangen-Nürnberg (FAU), Erlangen, Germany; ^2^Institut für Statistik, Ludwig-Maximilians-Universität München, Munich, Germany; ^3^Paul-Ehrlich-Institut, Langen, Germany

## Abstract

Statistical boosting algorithms have triggered a lot of research during the last decade. They combine a powerful machine learning approach with classical statistical modelling, offering various practical advantages like automated variable selection and implicit regularization of effect estimates. They are extremely flexible, as the underlying base-learners (regression functions defining the type of effect for the explanatory variables) can be combined with any kind of loss function (target function to be optimized, defining the type of regression setting). In this review article, we highlight the most recent methodological developments on statistical boosting regarding variable selection, functional regression, and advanced time-to-event modelling. Additionally, we provide a short overview on relevant applications of statistical boosting in biomedicine.

## 1. Introduction

Statistical boosting algorithms are one of the advanced methods in the toolbox of a modern statistician or data scientist [1]. While still yielding classical statistical models with well-known interpretability, they offer multiple advantages in the presence of high-dimensional data as they are applicable in *p* > *n* situations with more explanatory variables than observations [2, 3]. Key features in this context are automated variable selection and model choice [4, 5].

The research field embraces the world of statistics and computer science, bridging the gap between two rather different points of view on how to extract information from data [6]: on the one hand, there is the classical statistical modelling community who focus on models* describing* and* explaining* the outcome to find an approximation to the underlying stochastic data generating process. On the other hand, there is the machine learning community who focus primarily on algorithmic models* predicting* the outcome while treating the nature of the underlying process as unknown. Statistical boosting algorithms have their roots in machine learning [7] but were later adapted to estimate classical statistical models [8, 9]. A pivotal aspect of these algorithms is that they incorporate data-driven variable selection and shrinkage of effect estimates similar to classical penalized regression [10].

In a review some years ago [1], we highlighted this evolution of boosting from machine learning to statistical modelling. Furthermore, we emphasized the similarity of two boosting approaches, gradient boosting [2] and likelihood-based boosting [3], introducing* statistical boosting* as a generic term for these algorithms.

An accompanying article [11] highlighted the multiple extension of the basic algorithms towards (i) enhanced variable selection properties, (ii) new types of predictor effects, and (iii) new regression settings. Substantial methodological developments on statistical boosting algorithms throughout the last few years (e.g., stability selection [12]) and a growing community have opened the door to new model classes and frameworks (e.g., joint models [13] and functional data [14]), asking for an up-to-date review on the available extensions.

This article is structured as follows: In Section 2 we shortly highlight both basic structure and properties of statistical boosting algorithms and point to their connections to classical penalization approaches such as the lasso. In Section 3 we focus on new developments regarding variable selection (including exemplary analysis of gene expression data), which can also be combined with boosted functional regression models presented in Section 4.  Section 5 focuses on advanced survival models such as joint modelling; in Section 6 we briefly summarize other relevant developments and applications in the framework of statistical boosting.

## 2. Statistical Boosting

### 2.1. From Machine Learning to Statistical Models

The original boosting concept by Schapire [15] and Freund [7] emerged from the field of supervised learning where typically a function is trained based on data with known outcome classes or labels to correctly classify new observations. The aim of the boosting concept is to* boost* (i.e., to improve) the accuracy of weak classifiers (i.e., classifiers with poor correct classification rates) by iteratively applying them to reweighted data. Even if these so called* base-learners* individually only slightly outperform random guessing, the ensemble solution can often be boosted to a perfect classification [16].

The introduction of AdaBoost [17] was the breakthrough for boosting in the field of supervised machine learning, allegedly leading Leo Breiman to praise its performance:* Boosting is the best off-the-shelf classifier in the world* [18].

The main target of classical machine learning approaches is predicting observations *y*_new_ of the outcome *Y* given one or more input variables **X** = {*X*_1_,…, *X*_*p*_}. The estimation of the prediction rule (also called generalization function) is based on an observed sample (*y*_1_, **x**_1_),…, (*y*_*n*_, **x**_*n*_). However, the focus is not on quantifying or describing the underlying data generating process, but on predicting ${\hat{y}}_{new}$ for new observations *x*_new_ as accurately as possible. As a consequence, many machine learning approaches (also including the original AdaBoost with trees or stumps as base-learners) can be regarded as black box prediction schemes. Although typically yielding accurate predictions [19], they do not offer much insight into the structure of the relationship between explanatory variables **X** and the outcome *Y*.

Statistical regression models on the other hand particularly aim at describing and explaining the underlying relationship in a structured way. Not only can the impact of single explanatory variables be quantified in terms of variable importance measures [20, 21], but also the actual effect of these variables is interpretable. The work of Friedman et al. [8, 9] laid the groundwork to understand the concept of boosting from a statistical perspective and to adapt the general idea in order to estimate statistical models.

### 2.2. General Model Structure

The aim of* statistical boosting* algorithms is to estimate and select the effects in structured additive regression models. The most important model class are generalized additive models (“GAM” [22]), where the conditional distribution of the response variable is assumed to follow an exponential family distribution. The expected response is modelled given the observed value **x** of one or more explanatory variables using a link function *g* as

(1) $$\begin{array}{c} g\left(\;E\left(\;Y\;|\;X=x\;\right)\;\right)=f\left(\;x\;\right). \end{array}$$

In the typical case of multiple explanatory variables, the function *f*(**x**), which is often called additive predictor, consists of the additive effects of the single predictors:

(2) $$\begin{array}{c} f\left(\;x\;\right)=\beta_{0}+f_{1}\left(\;x_{1}\;\right)+\cdots +f_{p}\left(\;x_{p}\;\right), \end{array}$$

where *β*_0_ represents a common intercept and the functions *f*_*j*_(*x*_*j*_), *j* = 1,…*p*, are the individual effects of the variables *x*_*j*_. The generic notation *f*_*j*_(*x*_*j*_) may comprise different types of predictor effects such as classical linear effects, *x*_*j*_*β*_*j*_, smooth nonlinear effects constructed via regression splines, spatial effects, or random effects of the explanatory variable *x*_*j*_, to name but a few.

In statistical boosting algorithms, like the two approaches described in the following sections, the different effects are estimated by separate base-learners *h*_1_(·),…, *h*_*p*_(·) (*componentwise boosting* [2]). These base-learners are typically the corresponding simple regression-type prediction functions; for a linear effect, the corresponding base-learner would be a simple linear model: *h*_*j*_(*x*_*j*_) = *β*_0_ + *β*_1_*x*_*j*_.

### 2.3. The Generic Structure of Statistical Boosting

For a generic overview on the structure of statistical boosting algorithms, see Box 1. The base-learners are applied one by one, and in every iteration only the best performing base-learner *j*^*∗*^ is selected to be updated. The final additive model is hence the sum of all selected base-learner fits.

The main tuning parameter is *m*_stop_, the number of boosting iterations that is carried out. In order to avoid overfitting and to ensure variable selection, the algorithm is typically stopped before convergence* (early stopping)*. The selection of *m*_stop_ is based on the predictive performance evaluated via cross-validation or resampling [23]. This early stopping leads to an implicit penalization [24], similar to the lasso (see Section 2.6).

### 2.4. Gradient Boosting

In gradient boosting [2, 8], the iterative procedure fits the base-learners *h*_1_(*x*_1_),…, *h*_*p*_(*x*_*p*_) one by one to the negative gradient of the loss function *ρ*(*y*, *f*(·)), evaluated at the previous iteration:

(3) $$\begin{array}{c} u^{\left[m\right]}={\left(\;-\frac{\partial }{\partial f}\rho {\left.\;\left(\;y_{i},f\left(\;x_{i}\;\right)\;\right)\;\right|}_{f={\hat{f}}^{\left[m-1\right]}\left(x_{i}\right)}\;\right)}_{i=1,\ldots ,n}. \end{array}$$

The loss function describes the discrepancy between the observed outcome *y* and the additive predictor *f*(**x**_*i*_) and is the target function that should be minimized to get an optimal fit. In case of GAMs, the loss function is typically the negative log-likelihood of the corresponding exponential family. For Gaussian distributed outcomes, this reduces to the *L*_2_ loss *ρ*(*y*, *f*(**x**)) = (*y* − *f*(**x**))^2^, where the gradient vector **u**^[*m*]^ is simply the vector of residuals *y* − *f*(**x**) from iteration *m* − 1 and boosting hence corresponds to refitting of residuals.

In each boosting iteration, only the best-fitting base-learner *h*_*j*^*∗*^_ is selected based on the residual sum of squares of the base-learner fit

(4) $$\begin{array}{c} j^{*\left[m\right]}=\underset{1\leq j\leq p}{\arg\min}\overset{n}{\underset{i=1}{\sum }}{\left(\;u_{i}^{\left[m\right]}-{\hat{h}}_{j}^{\left[m\right]}\left(\;x_{ij}\;\right)\;\right)}^{2}. \end{array}$$

Only this base-learner *h*_*j*^*∗*^_ is added to the current additive predictor *f*(·). In order to ensure small updates, only a small proportion of the base-learner fit (typically the step length is *ν* = 0.1 [2]) is actually added. Note that the base-learner *h*_*j*_(·) can be selected and updated various times; the partial effect of variable *x*_*j*_ is the sum of all corresponding base-learners that had been selected:

(5) $$\begin{array}{c} {\hat{f}}_{j}\left(\;x_{j}\;\right)=\underset{m}{\sum }\nu \cdot {\hat{h}}_{j}^{\left[m\right]}\left(\;x_{j}\;\right)I_{j=j*\left[m\right]}. \end{array}$$

This componentwise procedure of fitting the base-learners one by one to the current gradient of the loss function can be described as* gradient descent in function space* [25], where the function space is spanned by the base-learners. The algorithm effectively optimizes the loss function step by step, eventually converging to the minimum.

Gradient boosting is implemented in the add-on package* mboost* [26] for the open source programming environment R [27], providing a large number of preimplemented loss functions for various regression settings, as well as different base-learners to represent various types of effects (see [28] for an overview; recent updates are summarized in Appendix).

### 2.5. Likelihood-Based Boosting

Likelihood-based boosting [3, 29] is the other general approach in the framework of statistical boosting algorithms; it received much attention particularly in the context of high-dimensional biomedical data (see [11] and the references therein). Although it follows a very similar structure to gradient boosting (see Box 1), both approaches only coincide in special cases such as classical Gaussian regression via the *L*_2_ loss [1, 30]. In contrast to gradient boosting, the base-learners are directly estimated via optimizing the overall likelihood, using the additive predictor from the previous iteration as offset. In case of the *L*_2_ loss, this has a similar consequence as refitting the residuals.

In every step, the algorithm hence optimizes regression models as base-learners one by one by maximizing the likelihood (using one-step Fisher scoring), selecting only the base-learner *j*^*∗*^ which leads to the largest increase in the likelihood. In order to obtain small boosting steps, a quadratic penalty term is attached to this likelihood. This has a similar effect to multiplying the fitted base-learner by a small step length factor as in gradient boosting.

Likelihood-based boosting for generalized linear and additive regression models is provided by the R add-on package* GAMBoost* [31], and an adapted version for boosting Cox regression is provided with* CoxBoost* [32]. For a comparison of both statistical boosting approaches, that is, likelihood-based and gradient boosting in case of Cox proportional hazard models, we refer to [33].

### 2.6. Connections to *L*_1_-Regularization

Statistical boosting algorithms result in regularized models with shrunk effect estimates although they only apply implicit penalization [24] by stopping the algorithm before convergence. By performing regularization without the use of an explicit penalty term, boosting algorithms clearly differ from other direct regularization techniques like the* lasso* [34]. However, both approaches sometimes result in very similar models after being tuned to a comparable degree of regularization [10].

This close connection has been first noted between the lasso and* forward stagewise regression*, which can be viewed as special case of the gradient boosting algorithm (Box 1), and led, along with the development of* least angle regression* (LARS), to the formulation of the* positive cone condition* (PCC) [35].

If this condition holds, LARS, lasso, and forward stagewise regression coincide. Figuratively speaking, the PCC requires that all coefficient estimates monotonically increase or decrease with relaxing degree of regularization and applies, for example, to the case of low-dimensional settings with orthogonal *X*. It should be noted that the PCC is connected to the* diagonal dominance condition* for the inverse covariance matrix of *X*, which allows for a more convenient way to investigate the equivalence of these approaches in practice [36, 37].

Given that the solution of the lasso is optimal with respect to the *L*_1_-norm of the coefficient vector, these findings led to the notion of boosting as some “sort of *L*_1_-sparse” regularization technique [38], but it remained unclear which optimality constraints possibly apply to forward stagewise regression if the PCC is violated.

By extending **X** with a negative version of each variable and enforcing only positive updates in each iteration, Hastie et al. [39] demonstrated that forward stagewise regression always approximates the solution path of a similarly modified version of the lasso. From this perspective, they showed that forward stagewise regression minimizes the loss function subject to the *L*_1_*-arc-length*: This means that the travelled path of the coefficients is penalized (allowing as little overall changes in the coefficients as possible, regardless of their direction), whereas the *L*_1_-norm considers only the absolute sum of the current set of estimates.

In the same article, Hastie et al. [39] further showed that these properties hold for general convex loss functions and therefore apply not only to forward stagewise regression but also for the more general gradient boosting method (in case of logistic regression models as well as for many other generalized linear regression settings).

The consequence of these differing optimization constraints can be observed in the presence of strong collinearity, where the lasso estimates tend to be very unstable regarding different degrees of regularization while boosting approaches avoid too many changes in the coefficients as they consider the overall travelled path [10].

It has to be acknowledged, however, that direct regularization approaches as the lasso are applied more often in practice [38]. Statistical boosting, on the other hand, is far more flexible due to its modular nature allowing combining any base-learner with any type of loss function [10, 38].

## 3. Enhanced Variable Selection

Early stopping of statistical boosting algorithms via cross-validation approaches plays a vital role in ensuring a sparse model with optimal prediction performance on new data. Resampling, that is, random sampling of the data drawn without replacement, tends to result in sparser models compared to other sampling schemes [23], including the popular bootstrap [40]. By using base-learners of comparable complexity (in terms of degrees of freedom) selection bias can be strongly reduced [4]. The resulting models have optimal prediction accuracy on the test data. Yet, despite regularization the final models are often relatively rich [23].

### 3.1. Stability Selection

Meinshausen and Bühlmann [41] proposed a generic approach called stability selection to further refine the models and enhance sparsity. This approach was then transferred to boosting [12].

In general, stability selection can be combined with any variable selection approach and is particularly useful for high-dimensional data with many potential predictors. To assess how stable the selection of a variable is, *B* random subsets that comprise half of the data are drawn. On each of these subsets, the model is fitted until a predefined number of *q* base-learners are selected. Usually, *B* = 100 subsets are sufficient. Computing the relative frequencies of random subsamples in which specific base-learners were selected gives a notion of how stable the selection is with respect to perturbations of the data. Base-learners are considered to be of importance if the selection frequency exceeds a prespecified threshold level *π*_thr_ ∈ [0.5,1].

Meinshausen and Bühlmann [41] showed that this approach controls the per-family error rate (PFER); that is, it provides an upper bound for the expected number of false positive selections (*V*):

(6) $$\begin{array}{c} E\left(\;V\;\right)\leq \frac{q^{2}}{\left(2\pi_{thr}-1\right)p}, \end{array}$$

where *p* is the number of base-learners. This upper bound is rather conservative and hence was further refined by Shah and Samworth [42] for specific assumptions on the distribution of the selection frequencies. Stability selection with all available error bounds is implemented for a variety of modelling techniques in the R package** stabs** [43].

An important issue is the choice of the hyperparameters of stability selection. The choice of a fixed value of *q* should be made such that it is large enough to select all hypothetically influential variables [12, 44]. A sensible value for *q* should usually be smaller than or equal to the number of base-learners selected via early stopping with cross-validation.

In general, the size of *q* is of minor importance if it is in a sensible range. With fixed *q*, either the threshold *π*_thr_ can be chosen or, as can be seen from (6) using equality, the upper bound for the PFER can be prespecified and the threshold can be derived accordingly. The latter would be the preferred choice if error control is of major importance and the former if error control is just considered a byproduct (see, e.g., [44]). For an interpretation of the PFER, particularly with regard to standard error rates such as the per-comparison error rate or the familywise error rate, we refer to Hofner et al. [12]. Note that, for fixed *q*, it is computationally easy to change any of the other two parameters (*π*_thr_ or the upper bound for the PFER) as the resampling results can be reused [12].

The result of stability selection is not a new prediction model but a set of* stable* base-learners: In fact they might not reflect any model which can be derived with a specific penalty parameter using the original modelling approach. This means that, for boosting, no *m*_stop_ value might exist that results in a model with the stably selected base-learners. The provided set of stable base-learners is a fundamentally new solution and not necessarily one with a high prediction accuracy [44].

### 3.2. Extension and Application of Boosting with Stability Selection

Variable selection is particularly important in high-dimensional gene expression data and other large scale biomedical data sources. Recently, stability selection with boosting was successfully applied to select a small number of informative biomarkers for survival of breast cancer patients [44]. The model was derived based on a novel boosting approach that optimizes the concordance index [45, 46]. Hence, the resulting prediction rule was optimal with respect to its ability to discriminate between patients with longer and shorter survival, that is, its discriminatory power.

Thomas et al. [47] derived a modified algorithm for boosted generalized additive models for location, scale, and shape (GAMLSS [48]) to allow a combination of this very flexible model class with stability selection. The basic idea of GAMLSS is to model all parameters of the conditional distribution by their own additive predictor and associated link function. Extensive simulation studies showed that the new fitting algorithm leads to comparable models as the previous algorithm [49, 50] but is superior regarding the computational speed, especially in combination with cross-validation approaches. Furthermore, simulations showed that this algorithm can be successfully combined with stability selection to select sparser models identifying a smaller subset of truly informative variables from high-dimensional data. The algorithm is implemented in the R add-on package* gamboostLSS* [51].

### 3.3. Stability Selection for Gene Expression Data

In the following, we demonstrate the application of stability selection based on gradient boosting on three high-dimensional datasets comprising gene expression levels. This includes oligonucleotide arrays for colon cancer detection (with *n* = 62 observations and *p* = 2000 gene expression levels) [52], prediction of metastasis of breast carcinoma (*n* = 168, *p* = 2905) [53], and Riboflavin production by Bacillus subtilis (*n* = 71, *p* = 4088) [54]. All three datasets are publicly available via the R packages* datamicroarray* [55] and* hdi* [56].

Regarding the parameters needed to be specified for stability selection, we investigate two different error rates PFER ∈ {1,3} and a constant *q* = 20. For the sake of comparison, we additionally apply 25-fold bootstrap for variable selection, which is the default setting for cross-validation in* mboost*.

Table 1 shows the total number of variables selected by each method. It can be seen that stability selection considerably reduces the set of variables in comparison with 25-fold bootstrap. In addition, relaxing the error bound results in larger sets except for the data on breast carcinoma, where only 1 base-learner entered the stable set.

### 3.4. Further Approaches for Sparse Models

In order to construct risk prediction signatures on molecular data, such as DNA methylation, Sariyar et al. [57] proposed an adaptive likelihood-based boosting algorithm. The authors included a step size modification factor *c*_*f*_ which represents an additional tuning parameter, adaptively controlling the size of the updates. In case of sparse settings, the approach decreases shrinkage of effect estimates (by using a larger step length) leading to a smaller bias. In settings with larger numbers of informative variables, the approach allows fitting models with lower degree of sparsity when necessary by smaller updates. The modification factor *c*_*f*_ has to be selected together with *m*_stop_ via cross-validation or resampling on a two-dimensional grid.

Zhang et al. [58] argue that variable ranking in practice is more favourable than variable selection, as ranking allows easily applying a thresholding rule in order to identify a subset of informative variables. The authors implemented a pseudo-boosting approach, which is technically not based on statistical boosting but is adapted to rank and select variables for statistical models. Note that also stability selection can be seen as a variable ranking scheme based on their selection frequency, as its selection feature is only triggered by implementing the threshold *π*_thr_.

Another recent proposal is to incorporate shadow-variables* (probing)* which are permuted variants of the original predictors in the candidate model [59]. The statistical boosting algorithm is stopped, when the first shadow-variable is selected. This way the focus of the tuning procedure is effectively shifted from prediction accuracy towards selection accuracy, which could be a fast and promising procedure to ensure sparse models.

Following a gradient based approach, Huang et al. [60] adapted the sparse boosting approach by Bühlmann and Yu [61] in order to promote similarity of model sparsity structures in the integrative analysis of multiple datasets, which is an important topic regarding the trend towards big data.

## 4. Functional Regression

Due to technological developments, more and more data is measured continuously over time. Over the last years, a lot of methodological research focused on regression methods for this type of functional data. A groundbreaking work in this new and evolving field of statistics is provided by Ramsay and Silverman [62].

Functional regression models can contain either functional responses (defined on a continuous domain), functional covariates, or both. This leads basically to three different classes of functional regression models, that is, function-on-scalar (response is functional), scalar-on-function (functional explanatory variable), and function-on-function regression. For recent reviews on functional regression, see Greven and Scheipl [63] and Morris [64].

### 4.1. Boosting Functional Data

The first statistical boosting algorithm for functional regression, allowing for data-driven variable selection, was proposed by Brockhaus et al. [65]. The authors' approach focused on linear array models [66] providing a unified framework for all three settings outlined above. Since the general structure of their gradient boosting algorithm is similar to the one in Box 1, the resulting models still have the same form as in (2), only that the response *Y* and the covariates may be functions. The underlying functional partial effects *h*_*j*_(**x**) can be represented using tensor product basis

(7) $$\begin{array}{c} h_{j}\left(\;x\;\right)\left(\;t\;\right)=\left(\;b_{j}{\left(\;x\;\right)}^{⊤}⊗b_{Y}{\left(\;t\;\right)}^{⊤}\;\right)\theta_{j}, \end{array}$$

where *θ*_*j*_ is the vector of coefficients, *b*_*j*_ and *b*_*Y*_ are basis functions, and ⊗ denotes the Kronecker product.

This functional array model is limited in two ways: (i) the functional responses need to be measured on a common grid and (ii) covariates need to be constant over the domain of the response. As particularly the second assumption might often not be fulfilled in practice, Brockhaus et al. [14] soon thereafter proposed a general framework for boosting functional regression models avoiding this assumption and dropping the linear array structure.

This newer framework [14] comprises also all three model classes outlined above and particularly focuses on historical effects, where functional response and functional covariates are observed over the same time interval. The underlying assumption is that observations of the covariate affect the response only up to the corresponding time point *t*

(8) $$\begin{array}{c} E\left(\;Y\left(\;t\;\right)\;|\;X=x\;\right)=\overset{J}{\underset{j=1}{\sum }}\overset{t}{\underset{t_{1}}{\int }}x_{j}\left(\;s\;\right)\beta_{j}\left(\;s,t\;\right)ds, \end{array}$$

where *s* represents the time points the covariate was observed for. In other words, only the part of the covariate function lying in the past (not the future) can affect the present response. However, this is a sensible restriction in most practical applications.

Both approaches for boosting functional regression are implemented in the R add-on package* FDboost* [67], which relies on the fitting methods and infrastructure of* mboost*.

### 4.2. Extensions of Boosting Functional Regression

Boosting functional data can be combined with stability selection (see Section 3.1) to enhance the variable selection properties of the algorithm [14, 65].

The boosting approach for functional data has already been extended towards the model class of generalized additive models for location, scale, and shape (GAMLSS) for a scalar-on-function setting by Brockhaus et al. [68]. The functional approach was named signal regression models for location, scale, and shape [68]. The estimation via gradient boosting is based on the corresponding gamboostLSS algorithm for boosting GAMLSS [49, 50].

In an approach to analyse the functional relationship between bioelectrical signals like electroencephalography (EEG) and facial electromyography (EMG), Rügamer et al. [69] focused on extending the framework of boosting functional regression by incorporating factor-specific historical effects, similar to (8).

Although functional data analysis triggered a lot of methodological research, a recent systematic review by Ullah and Finch [70] revealed that the number of actual biomedical applications of functional data analysis in general and functional regression in particular is rather small. The authors argued that the potential benefits of these flexible models (like richer interpretation and more flexible structures) are not yet well understood by practitioners and that further efforts are necessary to promote the actual usage of these novel techniques.

## 5. Boosting Advanced Survival Models

Cox regression is still the dominant model class for boosting time-to-event data; see [33] for a comparison of two different boosting algorithms and [71] for different general approaches to estimate Cox models in the presence of high-dimensional data. However, over the last years several alternatives emerged [45, 46, 72]. In this section we will particularly focus on boosting joint models of time-to-event outcomes and longitudinal markers but will also briefly refer to other recent extensions.

### 5.1. Boosting Joint Models

The concept of joint modelling of longitudinal and time-to-event data [73] has found its way into the statistical literature in the last few years as it thoroughly addresses questions on continuous data recorded over time and event times related to this continuous data. Modelling those two processes independently leads to misspecified models prone to bias. There are various joint modelling approaches and thus also various different model equations based on different covariates, distributions, and covariance structures. The type we are going to refer to in this review is the following:

(9) yij=ηlxij+ηlsxi,tij+εijλt ∣ α,ηsxi,t,ηlsxi,t=λ0texp⁡ηsxi,t+αηlsxi,t,

where *y*_*ij*_ is the *j*th observation of the *i*th individual with *i* = 1,…, *n* and *j* = 1,…, *n*_*i*_ and *λ*(*t*∣*α*, *η*_s_(*x*_*i*_, *t*), *η*_ls_(*x*_*i*_, *t*)) is the hazard function for individual *i* at time point *t*. Both outcomes, the longitudinal measurement *y*_*i*_ and the time *t*_*i*_, recorded alongside with the censoring indicator *δ*_*i*_, are modelled based on two subpredictors each: one that is supposed to have an impact on only one of them (the longitudinal subpredictor *η*_l_(*x*_*ij*_) and the survival subpredictor *η*_s_(*x*_*ij*_, *t*)) and the other being shared by both parts of the model (the shared subpredictor *η*_ls_(*x*_*ij*_, *t*)). All those subpredictors are functions of different, possibly time-dependent variables *x*_*i*_. The type of model presented here does not include fixed time varying covariates for the survival part of the model; please note that those models do exist but are not implemented in the boosting framework yet. It however includes time itself and, just like most joint models, some type of random effects. The function *λ*_0_(*t*) is the baseline hazard. Most approaches for joint models are based on likelihood or Bayesian inference using the joint likelihood resulting as a product from the corresponding likelihoods of the above processes [74, 75]. Those approaches are, however, unable to conduct variable selection and cannot deal with high-dimensional data.

Waldmann et al. [13] suggested a boosting algorithm tackling these challenges. The model used in that paper is a reduced version of (9) in which no survival subpredictor is considered and a fixed baseline hazard *λ*_0_ is used. The algorithm is a version of the classical boosting algorithm as represented in Box 1, which is adapted to the special case of having to estimate a set of different subpredictors (similar to the GAMLSS framework [49]). The algorithm is therefore composed of three steps which are performed circularly. In the first step a regular boosting step to update the longitudinal subpredictor *η*_l_(*x*_*ij*_) is performed and the parameters of the shared subpredictor are treated as fixed. In the second step, the parameters of the longitudinal subpredictor are fixed and a boosting step for the shared subpredictor *η*_ls_(*x*_*ij*_) is conducted. The third step is a simple optimization step: based on the current values of the parameters in both subpredictors the likelihoods are optimized with respect to *λ*_0_, *σ*^2^, and *α* (cf. [76]). The number of iterations now depends on two stopping iterations which have to be optimized on a two-dimensional grid via cross-validation.

Waldmann et al. [13] showed that the benefits of boosting algorithm (automated variable selection and handling of *p* > *n* situations) can be transferred to joint modelling and hence lay the groundwork to further extended joint modelling approaches.

### 5.2. An Example of Boosting Joint Models

The example presented in the following is similar to the simulation study in [13]. The simulated data consists of *N* = 500 individuals and a maximum of *n*_*i*_ = 5 observations per individual. Some observations are however truncated due to the risk function induced by the survival part of the model. The actual number of observations hence was 2350. The longitudinal subpredictor contains two informative variables and the intercept (*β*_l(0,1,2)_ = (2,1, −2)) as well as 1250 noninformative variables. The shared subpredictor has two fixed time invariant variables (*β*_ls(1,2)_ = (1, −2)), a time effect (*β*_*t*_ = 1), random intercept and slope, and also 1250 noninformative variables. In total there are hence 2508 covariates for 2350 observations, a situation clearly infeasible for ordinary joint modelling approaches.

We ran the above presented algorithm on this simulated example. By tenfold cross-validation we found the optimal stopping iterations to be *m*_stop,l_ = 125 and *m*_stop,ls_ = 130. The algorithm was able to detect the informative variables and the resulting coefficients were close to the original values ${\hat{\beta }}_{l(0,1,2)}=(2.042,0.993,-1.999)$, *β*_ls(1,2,*t*)_ = (0.971, −1.980,0.876). The longitudinal subpredictor furthermore selected three and the shared subpredictor two noninformative variables; hence only 0.2% of the noninformative variables were selected, all of which had absolute values below 0.023. Those results are typical findings for simulations done with the package based on the code for the approach presented here. It is available in the R add-on package* JMboost* [77], currently on GitHub.

### 5.3. Other New Approaches on Boosting Survival Data

Reulen and Kneib [78] extended the framework of statistical boosting towards multistate models for patients exposed to competing risks (e.g., adverse events, recovery, death, or relapse). The approach is implemented in the* gamboostMSM* package [79], relying on the infrastructure of* mboost*. Möst and Hothorn [80] focused on boosting the patient-specific survivor function based on conditional transformation models [81] incorporating inverse probability of censoring weights [82].

When statistical boosting algorithms are used to estimate survival models, the motivation most often is the presence of high-dimensional data. De Bin et al. [83] investigated several approaches (including gradient boosting and likelihood-based boosting) to incorporate both clinical and high-dimensional omics data in prediction models.

Guo et al. [84] proposed a new adaptive likelihood-based boosting algorithm to fit Cox models, incorporating a direct lasso-type *L*_1_ penalization in the fitting process in order to avoid the inclusion of variables with small effect. The general motivation is similar to the step length modification factor proposed by Sariyar et al. [57]. In another approach, Sariyar et al. [85] combined a likelihood-based boosting approach for the Cox model with random forests in order to screen for interaction effects in high-dimensional data. Hieke et al. [86] combined likelihood-based boosting with resampling to identify prognostic SNPs in potentially small clinical cohorts.

## 6. New Frontiers and Applications

Also other new topics have been incorporated into the framework of statistical boosting, but not all of them can be presented in detail here. However, we want to give a short overview of the most relevant developments, many of which were actually motivated by biomedical applications.

Weinhold et al. [87] proposed to analyse DNA methylation data (signal intensities *M* and *U*), via a “ratio of correlated gammas” model. Based on a bivariate gamma distribution for *M* and *U* values, the authors derived the density for the ratio *M*/(*M* + *U*) and optimized it via gradient boosting.

A boosting algorithm for differential item functioning in Rasch models was developed by Schauberger and Tutz [88] for the broader area of psychometrics, while Casalicchio et al. focused on boosting subject-specific Bradley-Terry-Luce models [89].

Napolitano et al. [90] developed a sampled boosting algorithm for the analysis of brain perfusion images: Gradient boosting is carried out multiple times on different training sets. Each base-learner refers to a voxel and after every sampling iteration a fixed fraction of selected voxels is randomly left out from the following boosting fit, to force the algorithm to select new voxels. The final model is then computed as the global sum of all solutions. Feilke et al. [91] proposed a voxelwise boosting approach for the analysis of contrast-enhanced magnetic resonance imaging data (DCE-MRI), which was additionally enhanced by a spatial penalty to account for the regional structure of the voxels.

Pybus et al. [92] proposed a hierarchical boosting algorithm for classification in an approach to detect positive selection in genomic regions (cf. [93]). Truntzer et al. [94] compared the classification performance of gradient boosting with other methods combining clinical variables and high-dimensional mass spectrometry data and concluded that the variable selection properties of boosting also led to a very good performance regarding prediction accuracy.

Regarding boosting location and scale models (modelling both expected value and variance in the spirit of GAMLSS [48]), Messner et al. [95] proposed a boosting algorithm for predictor selection in ensemble postprocessing to better calibrate ensemble weather forecasts. The idea of ensemble forecasting is to account for model errors and to quantify forecast uncertainty. Mayr et al. [96] used boosted location and scale models in combination with permutation tests to assess simultaneously systematic bias and random measurement errors of medical devices. The use of a permutation test tackles one of the remaining problems of statistical boosting approaches in practical biomedical research: The lack of standard errors for effect estimates makes it necessary to incorporate resampling procedures to construct confidence intervals or to assess significance of effects.

The methodological development in [96] was motivated by the analysis of biomedical data. Statistical boosting algorithms, however, have been applied over the last few years in various biomedical applications without the need for methodological extensions. Most applications focus on prediction modelling or variable selection.

To give an idea of the variety of topics, we briefly mention a selection of the most recent ones from the last two years. These applications comprise the development of birth weight prediction formulas for particularly small babies [97], prediction of smoking cessation and its relapse in HIV-infected patients [98],* Escherichia coli* Fed-Batch Fermentation Modelling [99], prediction of cardiovascular death for older patients in the emergency department [100], and identification of factors influencing therapeutic decisions regarding rheumatoid arthritis [101].

## 7. Discussion

In this article, we have highlighted several new research areas in the field of statistical boosting leaving the traditional GAM modelling approach. A particularly active research area during the last few years addresses the development of boosting algorithms for new model classes extending the GAM framework. These include, among others, the simultaneous modelling of location, scale, and shape parameters within the GAMLSS framework [49], the modelling of functional data [65], and, recently, the class of joint models for longitudinal and survival data [13]. It goes without saying that these developments will make boosting algorithms available for practical use in much more sophisticated clinical and epidemiological applications.

Another line of research aims at exploring the connections between statistical boosting methods and machine learning techniques that were originally developed independently of boosting. An important example is stability selection, a generic methodology that, at the time of its development, mainly focused on penalized regression models such as the lasso. Only recently has stability selection been adapted to become a tool for variable selection within the boosting framework (e.g., [47]). Other work in this context is the analysis of the connections between boosting and penalized regression [10] and the work by Sariyar et al. [85] exploring a combination of boosting and random forest methods.

Finally, as already noted by Hothorn [24], boosting may be regarded not only as a framework for regularized model fitting but also as a generic optimization tool in its own right. In particular, boosting constitutes a robust algorithm for the optimization of objective functions that, due to their structure or complexity, may pose problems for Newson-Raphson-type and related methods. This motivated the use of boosting in the articles by Hothorn et al. [81] and Weinhold et al. [87].

Regarding future research, a huge challenge for the use of boosting algorithms in biomedical applications arises from the* era of big data*. Unlike other machine learning methods like random forests, the sequential nature of boosting methods hampers the use of parallelization techniques within the algorithm, which may result in issues with the fitting and tuning of complex models with multidimensional predictors and/or sophisticated base-learners like splines or higher-sized trees. To overcome these problems in classification and univariate regression, Chen and Guestrin [102] developed the extremely fast and sophisticated* xgboost* environment.

For the more recent extensions discussed in this paper, however,* big data* solutions for statistical boosting have yet to be developed.

## Figures and Tables

**Box 1:**
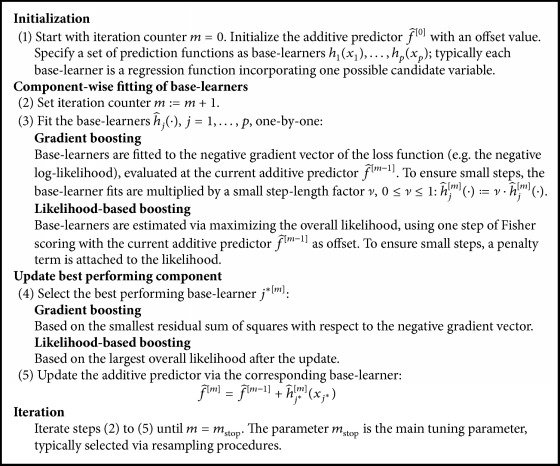
The structure of statistical boosting algorithms.

**Table 1:** Number of variables considered to be informative in different scenarios of stability selection and the default 25-fold bootstrap tuning of mboost without stability selection for comparison.

	Colon cancer	Breast carcinoma	Riboflavin production
PFER = 1, *q* = 20	2	1	4
PFER = 3, *q* = 20	3	1	5
25-fold bootstrap	11	28	39

## Appendix

### Developments regarding the mboost Package

This appendix describes important changes during the last years that were implemented in the R package* mboost* after the tutorial paper [28] on its use was published.

Starting from* mboost 2.2*, the default for the degrees of freedom was changed; they are now defined as

(A.1) $$\begin{array}{c} df\left(\;\lambda \;\right)=trace\left(\;2S-S^{⊤}S\;\right), \end{array}$$

with smoother matrix *S* = *X*(*X*^*⊤*^*X* + *λK*)^−1^*X*. Analyses have shown that this leads to a reduced selection bias; see [4]. Earlier versions used the trace of the smoother matrix as degrees of freedom; that is, df(*λ*) = trace(*S*). One can change to the old definition by setting options(mboost_dftraceS = TRUE). For parallel computations of cross-validated stopping values,* mboost* now uses the package* parallel*, which is included in the standard R installation. The behavior of bols(x, intercept = FALSE) was changed when x is a factor: the intercept is simply dropped from the design matrix and the coding can be specified as usual for factors. Additionally, a new contrast was introduced: "contr.dummy" (see the manual of bols for details). Finally, the computation of B-spline basis at the boundaries was changed such that equidistant boundary knots are used per default.

With* mboost 2.3*, constrained effects [103, 104] are fitted per default using quadratic programming methods (option type = "quad.prog") improving the speed of computation drastically. In addition to monotonic, convex, and concave effects, new constraints were introduced to fit "positive" or "negative" effects or effects with boundary constraints (see bmono for details). Additionally, a new function to assign *m*_stop_ values to a model object was added (mstop(mod) <- i) as well as two new distribution families Hurdle [105] and Multinomial [76]. Finally, a new option was implemented to allow for stopping based on out-of-bag data during fitting (via boost_control(…, stopintern = TRUE)).

With* mboost 2.4*, bootstrap confidence intervals were implemented in the novel confint function [104]. The stability selection procedure was moved to a dedicated package** stabs** [43], while a specific function for gradient boosting was implemented in package* mboost*.

From* mboost 2.5* onward, cross-validation does not stop on errors in single folds anymore and was sped up by setting mc.preschedule = FALSE if parallel computations via mclapply are used. A documentation for the function plot.mboost was added, which allows visualizing model results. Values outside the boundary knots are now forbidden during fitting, while linear extrapolation is used for prediction.

With* mboost 2.6* a lot of bug fixes and small improvements were provided. Most notably, the development of the package is now hosted entirely on github in the collaborative project boost-R/mboost and the package maintainer changed.

The* mboost 2.7* version provides a new family Cindex [45], variable importance measures (varimp), and improved plotting facilities.

The current CRAN version* mboost 2.8* includes major changes to the Binomial family which now additionally provides an alternative implementation of Binomial regression models along the lines of the classic glm implementation, which can be used via Binomial(type = "glm"). This family also works with a two-column matrix containing the number of successes and number of failures. Furthermore, models with zero steps (i.e., models containing only the offset) are supported and cross-validation can now select models without base-learners. Finally, a new base-learner bkernel for pathway-based kernel boosting in genome-wide association studies (GWAS) was added [106].

## References

[1] Mayr A., Binder H., Gefeller O., Schmid M. (2014). The evolution of boosting algorithms: from machine learning to statistical modelling. *Methods of Information in Medicine*.

[2] Bühlmann P., Hothorn T. (2007). Rejoinder: boosting algorithms: regularization, prediction and model fitting. *Statistical Science*.

[3] Tutz G., Binder H. (2006). Generalized additive modeling with implicit variable selection by likelihood-based boosting. *Biometrics*.

[4] Hofner B., Hothorn T., Kneib T., Schmid M. (2011). A framework for unbiased model selection based on boosting. *Journal of Computational and Graphical Statistics*.

[5] Kneib T., Hothorn T., Tutz G. (2009). Variable selection and model choice in geoadditive regression models. *Biometrics*.

[6] Breiman L. (2001). Statistical modeling: the two cultures. *Statistical Science*.

[7] Freund Y., Fulk M. A., Case J. Boosting a weak learning algorithm by majority.

[8] Friedman J. H. (2001). Greedy function approximation: a gradient boosting machine. *The Annals of Statistics*.

[9] Friedman J., Hastie T., Tibshirani R. (2000). Additive logistic regression: a statistical view of boosting. *The Annals of Statistics*.

[10] Hepp T., Schmid M., Gefeller O., Waldmann E., Mayr A. (2016). Approaches to regularized regression - A comparison between gradient boosting and the lasso. *Methods of Information in Medicine*.

[11] Mayr A., Binder H., Gefeller O., Schmid M. (2014). Extending statistical boosting. *Methods of Information in Medicine*.

[12] Hofner B., Boccuto L., Göker M. (2015). Controlling false discoveries in high-dimensional situations: Boosting with stability selection. *BMC Bioinformatics*.

[13] Waldmann E., Taylor-Robinson D., Klein N. (2017). Boosting joint models for longitudinal and time-to-event data. *Biometrical Journal*.

[14] Brockhaus S., Melcher M., Leisch F., Greven S. (2017). Boosting flexible functional regression models with a high number of functional historical effects. *Statistics and Computing*.

[15] Schapire R. E. (1990). The strength of weak learnability. *Machine Learning*.

[16] Schapire R., Freund Y. (2012). *Boosting: Foundations and Algorithms*.

[17] Freund Y., Schapire R. Experiments with a new boosting algorithm.

[18] Hastie T., Tibshirani R., Friedman J. (2009). *The Elements of Statistical Learning: Data Mining, Inference, and Prediction*.

[19] Wyner A. J., Olson M., Bleich J., Mease D. (2017). Explaining the success of adaboost and random forests as interpolating classifiers. *Journal of Machine Learning Research*.

[20] Strobl C., Boulesteix A.-L., Zeileis A., Hothorn T. (2007). Bias in random forest variable importance measures: illustrations, sources and a solution. *BMC Bioinformatics*.

[21] Hapfelmeier A., Hothorn T., Ulm K., Strobl C. (2014). A new variable importance measure for random forests with missing data. *Statistics and Computing*.

[22] Hastie T. J., Tibshirani R. J. (1990). *Generalized Additive Models*.

[23] Mayr A., Hofner B., Schmid M. (2012). The importance of knowing when to stop: a sequential stopping rule for component-wise gradient boosting. *Methods of Information in Medicine*.

[24] Hothorn T. (2014). Boosting – An unusual yet attractive optimiser. *Methods of Information in Medicine*.

[25] Mason L., Baxter J., Bartlett P., Frean M. Boosting algorithms as gradient descent.

[26] Hothorn T., Bühlmann P., Kneib T., Schmid M., Hofner B. mboost: Model-Based Boosting. https://CRAN.R-project.org/package=mboost.

[27] R Development Core Team (2016). *R: A Language and Environment for Statistical Computing*.

[28] Hofner B., Mayr A., Robinzonov N., Schmid M. (2014). Model-based boosting in R: a hands-on tutorial using the R Package mboost. *Computational Statistics*.

[29] Tutz G., Binder H. (2007). Boosting ridge regression. *Computational Statistics and Data Analysis*.

[30] Bühlmann P., Yu B. (2003). Boosting with the L2 loss: regression and classification. *Journal of the American Statistical Association*.

[31] Binder H. (2011). *GAMBoost: Generalized Linear and Additive Models by Likelihood Based Boosting*.

[32] Binder H. (2013). *CoxBoost: Cox Models by Likelihood-based Boosting for a Single Survival Endpoint or Competing Risks*.

[33] De Bin R. (2016). Boosting in Cox regression: a comparison between the likelihood-based and the model-based approaches with focus on the R-packages CoxBoost and mboost. *Computational Statistics*.

[34] Tibshirani R. (1996). Regression shrinkage and selection via the lasso. *Journal of the Royal Statistical Society - Series B*.

[35] Efron B., Hastie T., Johnstone I., Tibshirani R. (2004). Least angle regression. *The Annals of Statistics*.

[36] Meinshausen N., Rocha G., Yu B. (2007). Discussion: a tale of three cousins: Lasso, L2 boosting and Dantzig. *The Annals of Statistics*.

[37] Duan J., Soussen C., Brie D., Idier J., Wang Y.-P. (2012). On LARS/homotopy equivalence conditions for over-determined LASSO. *IEEE Signal Processing Letters*.

[38] Bühlmann P., Gertheiss J., Hieke S. (2014). Discussion of 'the evolution of boosting algorithms' and 'extending statistical boosting'. *Methods of Information in Medicine*.

[39] Hastie T., Taylor J., Tibshirani R., Walther G. (2007). Forward stagewise regression and the monotone lasso. *Electronic Journal of Statistics*.

[40] Janitza S., Binder H., Boulesteix A.-L. (2016). Pitfalls of hypothesis tests and model selection on bootstrap samples: causes and consequences in biometrical applications. *Biometrical Journal*.

[41] Meinshausen N., Bühlmann P. (2010). Stability selection. *Journal of the Royal Statistical Society Series B*.

[42] Shah R. D., Samworth R. J. (2013). Variable selection with error control: another look at stability selection. *Journal of the Royal Statistical Society. Series B. Statistical Methodology*.

[43] Hofner B., Hothorn T., stabs. (2017). *Stability Selection with Error Control*.

[44] Mayr A., Hofner B., Schmid M. (2016). Boosting the discriminatory power of sparse survival models via optimization of the concordance index and stability selection. *BMC Bioinformatics*.

[45] Mayr A., Schmid M. (2014). Boosting the concordance index for survival data - a unified framework to derive and evaluate biomarker combinations. *PLoS ONE*.

[46] Chen Y., Jia Z., Mercola D., Xie X. (2013). A gradient boosting algorithm for survival analysis via direct optimization of concordance index. *Computational and Mathematical Methods in Medicine*.

[47] Thomas J., Mayr A., Bischl B., Schmid M., Smith A., Hofner B. (2017). Gradient boosting for distributional regression: faster tuning and improved variable selection via noncyclical updates. *Statistics and Computing*.

[48] Rigby R. A., Stasinopoulos D. M. (2005). Generalized additive models for location, scale and shape. *Journal of the Royal Statistical Society. Series C. Applied Statistics*.

[49] Mayr A., Fenske N., Hofner B., Kneib T., Schmid M. (2012). Generalized additive models for location, scale and shape for high dimensional data---a flexible approach based on boosting. *Journal of the Royal Statistical Society. Series C. Applied Statistics*.

[50] Hofner B., Mayr A., Schmid M. (2016). gamboostLSS: an R package for model building and variable selection in the GAMLSS framework. *Journal of Statistical Software*.

[51] Hofner B., Mayr A., Fenske N., Thomas J., Schmid M. gamboostLSS: Boosting Methods for GAMLSS Models. https://CRAN.R-project.org/package=gamboostLSS.

[52] Alon U., Barka N., Notterman D. A. (1999). Broad patterns of gene expression revealed by clustering analysis of tumor and normal colon tissues probed by oligonucleotide arrays. *Proceedings of the National Academy of Sciences of the United States of America*.

[53] Gravier E., Pierron G., Vincent-Salomon A. (September 2009). A prognostic DNA signature for T1T2 node-negative breast cancer patients. *Genes Chromosomes and Cancer*.

[54] Bühlmann P., Kalisch M., Meier L. (2014). High-dimensional statistics with a view toward applications in Biology. *Annual Review of Statistics and Its Application*.

[55] Ramey J. A. Datamicroarray: Collection of Data Sets for Classification. https://github.com/ramhiser/datamicroarray.

[56] Dezeure R., Bühlmann P., Meier L., Meinshausen N. (2015). High-dimensional inference: confidence intervals, *p*-values and R-software hdi. *Statistical Science*.

[57] Sariyar M., Schumacher M., Binder H. (2014). A boosting approach for adapting the sparsity of risk prediction signatures based on different molecular levels. *Statistical Applications in Genetics and Molecular Biology*.

[58] Zhang C.-X., Zhang J.-S., Kim S.-W. (2016). PBoostGA: pseudo-boosting genetic algorithm for variable ranking and selection. *Computational Statistics*.

[59] Thomas J., Hepp T., Mayr A., Bischl B. Probing for sparse and fast variable selection with model-based boosting.

[60] Huang Y., Liu J., Yi H., Shia B.-C., Ma S. (2017). Promoting similarity of model sparsity structure in integrative analysis of cancer genetic data. *Statistics in Medicine*.

[61] Bühlmann P., Yu B. (2006). Sparse boosting. *Journal of Machine Learning Research*.

[62] Ramsay J. O., Silverman B. W. (2002). *Applied functional data analysis: methods and case studies*.

[63] Greven S., Scheipl F. (2017). A general framework for functional regression modelling. *Statistical Modelling*.

[64] Morris J. S. (2015). Functional regression. *Annual Review of Statistics and Its Application*.

[65] Brockhaus S., Scheipl F., Hothorn T., Greven S. (2015). The functional linear array model. *Statistical Modelling*.

[66] Currie I. D., Durban M., Eilers P. H. (2006). Generalized linear array models with applications to multidimensional smoothing. *Journal of the Royal Statistical Society. Series B. Statistical Methodology*.

[67] Brockhaus S., Rügamer D. (2016). *Brockhaus S, Rügamer D. FDboost: Boosting Functional Regression Models; R package version 0.2-0*.

[68] Brockhaus S., Fuest A., Mayr A., Greven S. Signal regression models for location, scale and shape with an application to stock returns.

[69] Rügamer D., Brockhaus S., Gentsch K., Scherer K., Greven S. Boosting factor-specific functional historical models for the detection of synchronisation in bioelectrical signals.

[70] Ullah S., Finch C. F. (2013). Applications of functional data analysis: a systematic review. *BMC Medical Research Methodology*.

[71] Zemmour C., Bertucci F., Finetti P. (2015). Prediction of early breast cancer metastasis from dna microarray data using high-dimensional Cox regression models. *Cancer Informatics*.

[72] Schmid M., Hothorn T. (2008). Flexible boosting of accelerated failure time models. *BMC Bioinformatics*.

[73] Wulfsohn M. S., Tsiatis A. A. (1997). A joint model for survival and longitudinal data measured with error. *Biometrics*.

[74] Faucett C. L., Thomas D. C. (1996). Simultaneously modelling censored survival data and repeatedly measured covariates: a Gibbs sampling approach. *Statistics in Medicine*.

[75] Rizopoulos D. (2010). JM: an R package for the joint modelling of longitudinal and time-to-event data. *Journal of Statistical Software*.

[76] Schmid M., Potapov S., Pfahlberg A., Hothorn T. (2010). Estimation and regularization techniques for regression models with multidimensional prediction functions. *Statistics and Computing*.

[77] Waldmann E., Mayr A. JMboost: Boosting Joint Models for Longitudinal and Time-to-Event Outcomes. https://github.com/mayrandy/JMboost.

[78] Reulen H., Kneib T. (2016). Boosting multi-state models. *Lifetime Data Analysis*.

[79] Reulen H. gamboostMSM: Estimating multistate models using gamboost(). https://CRAN.R-project.org/package=gamboostMSM.

[80] Möst L., Hothorn T. (2015). Conditional transformation models for survivor function estimation. *The International Journal of Biostatistics*.

[81] Hothorn T., Kneib T., Bühlmann P. (2014). Conditional transformation models. *Journal of the Royal Statistical Society. Series B. Statistical Methodology*.

[82] van der Laan M. J., Robins J. M. (2003). *Unified Methods for Censored Longitudinal Data and Causality*.

[83] De Bin R., Sauerbrei W., Boulesteix A.-L. (2014). Investigating the prediction ability of survival models based on both clinical and omics data: two case studies. *Statistics in Medicine*.

[84] Guo Z., Lu W., Li L. (2015). Forward Stagewise Shrinkage and Addition for High Dimensional Censored Regression. *Statistics in Biosciences*.

[85] Sariyar M., Hoffmann I., Binder H. (2014). Combining techniques for screening and evaluating interaction terms on high-dimensional time-to-event data. *BMC Bioinformatics*.

[86] Hieke S., Benner A., Schlenk R. F., Schumacher M., Bullinger L., Binder H. (2016). Identifying prognostic SNPs in clinical cohorts: complementing univariate analyses by resampling and multivariable modeling. *PLoS ONE*.

[87] Weinhold L., Wahl S., Pechlivanis S., Hoffmann P., Schmid M. (2016). A statistical model for the analysis of beta values in DNA methylation studies. *BMC Bioinformatics*.

[88] Schauberger G., Tutz G. (2016). Detection of differential item functioning in Rasch models by boosting techniques. *British Journal of Mathematical and Statistical Psychology*.

[89] Casalicchio G., Tutz G., Schauberger G. (2015). Subject-specific Bradley-Terry-Luce models with implicit variable selection. *Statistical Modelling*.

[90] Napolitano G., Stingl J. C., Schmid M., Viviani R. (2017). Predicting CYP2D6 phenotype from resting brain perfusion images by gradient boosting. *Psychiatry Research: Neuroimaging*.

[91] Feilke M., Bischl B., Schmid V. J., Gertheiss J. (2016). Boosting in nonlinear regression models with an application to DCE-MRI data. *Methods of Information in Medicine*.

[92] Pybus M., Luisi P., Dall'Olio G. M. (2015). Hierarchical boosting: a machine-learning framework to detect and classify hard selective sweeps in human populations. *Bioinformatics*.

[93] Lin K., Li H., Schlötterer C., Futschik A. (2011). Distinguishing positive selection from neutral evolution: Boosting the performance of summary statistics. *Genetics*.

[94] Truntzer C., Mostacci E., Jeannin A., Petit J.-M., Ducoroy P., Cardot H. (2014). Comparison of classification methods that combine clinical data and high-dimensional mass spectrometry data. *BMC Bioinformatics*.

[95] Messner J. W., Mayr G. J., Zeileis A. (2017). Nonhomogeneous Boosting for Predictor Selection in Ensemble Postprocessing. *Monthly Weather Review*.

[96] Mayr A., Schmid M., Pfahlberg A., Uter W., Gefeller O. (2017). A permutation test to analyse systematic bias and random measurement errors of medical devices via boosting location and scale models. *Statistical Methods in Medical Research*.

[97] Faschingbauer F., Dammer U., Raabe E. (2016). A new sonographic weight estimation formula for small-for-gestational-age fetuses. *Journal of Ultrasound in Medicine*.

[98] Schäfer J., Young J., Bernasconi E. (2015). Predicting smoking cessation and its relapse in HIV-infected patients: the swiss HIV cohort study. *HIV Medicine*.

[99] Melcher M., Scharl T., Luchner M., Striedner G., Leisch F. (2017). Boosted structured additive regression for. *Biotechnology and Bioengineering*.

[100] Bahrmann P., Christ M., Hofner B. (2016). Prognostic value of different biomarkers for cardiovascular death in unselected older patients in the emergency department. *European Heart Journal: Acute Cardiovascular Care*.

[101] Pattloch D., Richter A., Manger B. (2017). Das erste Biologikum bei rheumatoider arthritis: einflussfaktoren auf die Therapieentscheidung. *Zeitschrift für Rheumatologie*.

[102] Chen T., Guestrin C. XGBoost: a scalable tree boosting system.

[103] Hofner B., Müller J., Hothorn T. (2011). Monotonicity-constrained species distribution models. *Ecology*.

[104] Hofner B., Kneib T., Hothorn T. (2016). A unified framework of constrained regression. *Statistics and Computing*.

[105] Hofner B., Smith A. Boosted negative binomial hurdle models for spatiotemporal abundance of sea birds.

[106] Friedrichs S., Manitz J., Burger P. (2017). Pathway-based kernel boosting for the analysis of genome-wide association studies. *Computational and Mathematical Methods in Medicine*.

